# Paracrine Effects of Bone Marrow Mononuclear Cells in Survival and Cytokine Expression after 90% Partial Hepatectomy

**DOI:** 10.1155/2017/5270527

**Published:** 2017-02-23

**Authors:** Carlos Oscar Kieling, Carolina Uribe-Cruz, Mónica Luján López, Alessandro Bersch Osvaldt, Themis Reverbel da Silveira, Ursula Matte

**Affiliations:** ^1^Experimental Laboratory of Gastroenterology and Hepatology, HCPA, Ramiro Barcelos 2350, 90035-903 Porto Alegre, RS, Brazil; ^2^Post-Graduation Program on Medicine: Sciences in Gastroenterology, UFRGS, Ramiro Barcelos 2350, 90035-903 Porto Alegre, RS, Brazil; ^3^Gene Therapy Center, HCPA, Ramiro Barcelos 2350, 90035-903 Porto Alegre, RS, Brazil; ^4^Post-Graduation Program on Genetics and Molecular Biology, UFRGS, Av. Bento Gonçalves 9500, 91501-970 Porto Alegre, RS, Brazil; ^5^Post-Graduation Program in Surgery, UFRGS, Ramiro Barcelos 2350, 90035-903 Porto Alegre, RS, Brazil; ^6^Post-Graduation Program in Child and Adolescent Health, UFRGS, Ramiro Barcelos 2350, 90035-903 Porto Alegre, RS, Brazil

## Abstract

Acute liver failure is a complex and fatal disease. Cell-based therapies are a promising alternative therapeutic approach for liver failure due to relatively simple technique and lower cost. The use of semipermeable microcapsules has become an interesting tool for evaluating paracrine effects in vivo. In this study, we aimed to assess the paracrine effects of bone marrow mononuclear cells (BMMC) encapsulated in sodium alginate to treat acute liver failure in an animal model of 90% partial hepatectomy (90% PH). Encapsulated BMMC were able to increase 10-day survival without enhancing liver regeneration markers. Gene expression of* Il-6* and* Il-10 *in the remnant liver was markedly reduced at 6 h after 90% PH in animals receiving encapsulated BMMC compared to controls. This difference, however, was neither reflected by changes in the number of CD68+ cells nor by serum levels of IL6. On the other hand, treated animals presented increased caspase activity and gene expression in the liver. Taken together, these results suggest that BMMC regulate immune response and promote apoptosis in the liver after 90% PH by paracrine factors. These changes ultimately may be related to the higher survival observed in treated animals, suggesting that BMMC may be a promising alternative to treat acute liver failure.

## 1. Introduction

Acute liver failure is a complex and fatal disease, characterized by jaundice, coagulopathy, and hepatic encephalopathy [1]. The etiology varies from viral hepatitis, drug-induced hepatotoxicity, and metabolic liver disease to uncertain causes [1, 2]. Liver transplantation remains the only proven treatment for end-stage liver failure but is limited by the availability of donor organs [1, 3].

Cell-based therapies are a promising alternative therapeutic approach for liver failure due to relatively simple technique and lower cost [3, 4]. Several preclinical and clinical experiments have been reported on the safety and efficacy of bone-marrow-derived mononuclear cells to treat liver disorders [5–8].

Recently, one of the best studied mechanisms of action for these cells is the release of paracrine factors cells [9]. However, the pathway in which they act has still not been fully clarified mainly by the difficulty of in vivo studies. The use of semipermeable microcapsules has become an interesting tool for evaluating paracrine effects in vivo. These microcapsules can immune-isolate xenogeneic cells allowing the exchange of low-molecular-weight nutrients and oxygen across the membranes [10]. In this study, we aimed to assess the paracrine effect of bone marrow mononuclear cells (BMMC) encapsulated in sodium alginate to treat acute liver failure in an animal model of 90% partial hepatectomy.

## 2. Materials and Methods

### 2.1. Animals

Two-month-old male Wistar rats (298 ± 60 g) were kept under controlled temperature (between 18 and 22°C) in 12 h light-dark cycles with free access to water and standard chow at Experimental Animal Unit at Hospital de Clínicas de Porto Alegre (HCPA). Handling, care, and processing of animals were carried out according to regulations approved by our local Ethics Committee and complied with the National Guidelines on Animal Care.

### 2.2. Experimental Design

Rats were randomly divided into two groups. Treated group received encapsulated bone marrow mononuclear cells (BMMC, *n* = 39) and control group (*n* = 41) received empty capsules (EC). Survival was observed for up to 10 days after 90% PH.

To evaluate the early effects of treatments an additional set of animals were randomly divided into two groups (BMMC and EC) and euthanized at 6, 12, 24, 48, and 72 hours after 90% PH (*n* = 6–8/group/time point). For mononuclear bone marrow cells 5 animals were used as donors and another 5 animals without liver injury were used as normal controls.

### 2.3. Isolation of BMMC

Five naïve male Wistar rats were used as BMMC donors as described by Matte et al. [11]. Briefly, in a sterile environment, the femurs and tibias were isolated and whole bone marrow was flushed with complete medium: DMEM (Dulbecco's Modified Eagle Medium, LGC, Brazil) supplemented with 10% fetal bovine serum (Gibco, USA), 1% penicillin/streptomycin (Gibco, USA) and centrifuged at 800 g for 5 minutes. The pellet was diluted in complete medium and then placed onto a Ficoll Histopaque (GE-Healthcare, USA) layer and centrifuged at 800 g for 30 min. The interface was separated using a pipette, and cells were rinsed with PBS three times. Cells were counted using the Neubauer chamber and Trypan Blue exclusion test to verify cell viability.

### 2.4. Cell Encapsulation

Cell encapsulation was performed according to our laboratory protocol, previously described [12]. Briefly, BMMC were mixed with 1.5% sodium alginate (Sigma-Aldrich, USA) in complete medium and extruded through a Encapsulation Unit, type J1 (Nisco, Switzerland), attached to JMS Syringe Pump. Droplets were sheared off with an air flow of 5 L/min delivered to the tip of a 27 G needle and the rate of infusion was 40 mL/h. The droplets fell into a bath of 125 mM CaCl_2_ and ionically cross-linked with Ca_2_^+^ to form solid spherical hydrogel beads containing embedded BMMC. In each well capsules were produced from a volume of 2 mL of alginate suspension, containing 1 × 10^6^ BMMC/animal. BMMC encapsulation was carried out under sterile conditions. For control group, 2 mL of empty capsules was produced using the same approach, although without cells. The resulting capsules were maintained under normal cell culture conditions with complete medium at 37°C and 5% CO_2_ for 24 h prior to administration.

### 2.5. Animal Model of 90% Partial Hepatectomy and Capsules Transplantation

Hepatectomy was carried out under isoflurane (Forane®, Abbott SA, Argentina) anesthesia [13]. An abdominal midline incision was made to expose the liver. Ninety percent hepatectomy was performed by a single operator as described by Gaub and Iversen (1984) [14]. In brief, the left lateral (30%), left median (40%), and right superior lobes (20%) were removed, leaving only the caudate lobes. Immediately after 90% PH, as well as before complete suture, microcapsules (either empty or containing BMMC) were placed into the peritoneal cavity and glucose was supplemented i.p. (5% of body weight). The incision was then closed. After the rats recovered from anesthesia, animals were given i.p. glucose (5% of body weight) until day seven and received 20% glucose in their drinking water and standard chow ad libitum until euthanasia.

### 2.6. Euthanasia

Euthanasia was performed in CO_2_ chambers at the 6, 12, 24, 48, and 72 h or at 10 days after 90% PH. Immediately after euthanasia blood was collected and the serum was kept at −80°C until analysis; the liver was removed and weighed and part was flash-frozen in liquid nitrogen and the rest was placed in 10% buffered formalin. At each time point and every day until day 10 animals were weighed.

### 2.7. Liver Regeneration Rate

The liver regeneration rate [15] was calculated as follows: liver regeneration rate  (%) = 100 × [*C* − (*A* − *B*)]/*A*, where *A* is the estimated liver weight before PH, *B* is the excised liver weight at the time of PH, and *C* is the weight of the regenerated liver at the time of euthanasia.

### 2.8. Histology and Immunohistochemistry

Paraffin-embedded liver specimens were cut in 4 *μ*m sections and stained with hematoxylin and eosin (H-E). Mitotic index was performed by counting the number of hepatocytes undergoing mitosis in 10 high power fields (HPF) at each time point until 72 h after 90% PH [6].

To assess the rate of hepatocyte proliferation, the number of hepatocytes undergoing mitosis was counted in 10 HPF. In addition, 5-bromo-2′-deoxyuridine (BrdU) immunostaining was done using BrdU staining kit (Invitrogen, USA). Two hours before sacrifice, rats (*n* = 3/group) were injected with BrdU (1 mL/g). Thereafter, liver sections were incubated with BrdU antibody and the number of positive hepatocytes was counted in 5 HPF.

### 2.9. Serum Cytokine Levels

Serum level of IL-6 was quantified by enzyme-linked immunosorbent assay (ELISA) using commercial kits (R&D Systems®, Minneapolis, Minnesota, EUA) in accordance with the manufacturer's instructions.

### 2.10. Quantitative Real-Time PCR

Total RNA was extracted from liver tissue (~50 mg) using TRIzol reagent (Invitrogen, USA) according to the manufacturer's instructions and 2 *μ*g was reverse-transcribed using High Capacity cDNA Reverse Transcription Kit (Life Technologies, USA). The expression of genes involved in inflammation pathway (Interleukins 6 and 10) and apoptosis (Caspase 3) were measured using TaqMan® assays (Life Technologies, USA). The percentage of a test RNA to that of *β*-actin was calculated by subtracting the cycle to reach the threshold (CT) for that gene from the CT for *β*-actin to determine the ΔCT, and the formula is percent *β*-actin = (100) × 2ΔCT [12]. The percent *β*-actin for hepatectomized animals was divided by the percent *β*-actin in normal animals to determine the ratio of gene expression in both treatments after 90% PH to normal rats. Livers of animals without injury were used as calibrator group (normal values = 1).

### 2.11. Immunohistochemistry Analysis

Paraffin-embedded liver specimens were cut in 4 *μ*m sections and Kupffer cells were quantified by immunostaining for CD68. For that, liver sections were incubated overnight at 4°C with the primary antibody rabbit IgG-CD68 (1 : 800, Abcam, USA) and washed with phosphate buffer Tween 20; then universal biotinylated link and streptavidin-HRP were added (Dako, USA) and revealed with DAB kit (Dako, USA). The slides were counterstained with hematoxylin. The number of CD68^+^ cells was counted in 5 randomly selected HPF (×400) per slide.

### 2.12. Caspase 3 Activity

Fluorometric Caspase 3 activity (Sigma-Aldrich, USA) assays were performed according to the manufacturer's instructions. Briefly, 15 *μ*L of liver homogenate in PBS was placed in an opaque 96-well plate and 200 *μ*L of mixture reaction solution (containing Acetyl-Asp-Glu-Val-Asp-7-amido-4-methylcoumarin) was added to each well. The plate was incubated in dark at 25°C and every 10 minutes the fluorescence was read at 360 nm of excitation and 460 of emission. Caspase activity was normalized by protein measured by Lowry method [16].

### 2.13. Statistical Analysis

Results were expressed as means ± standard deviation (SD) or medians when required. Statistical differences were assessed by Student's *t*-test and for nonparametric variables Mann–Whitney test was used. The survival rate was analyzed by Kaplan-Meier curve. The comparison of survival rates in different groups was tested by the log rank test. *P* values less than 0.05 were considered statistically significant.

## 3. Results

### 3.1. BMMC Increase Survival Rate in Rats with 90% Partial Hepatectomy

**Figure 1:**
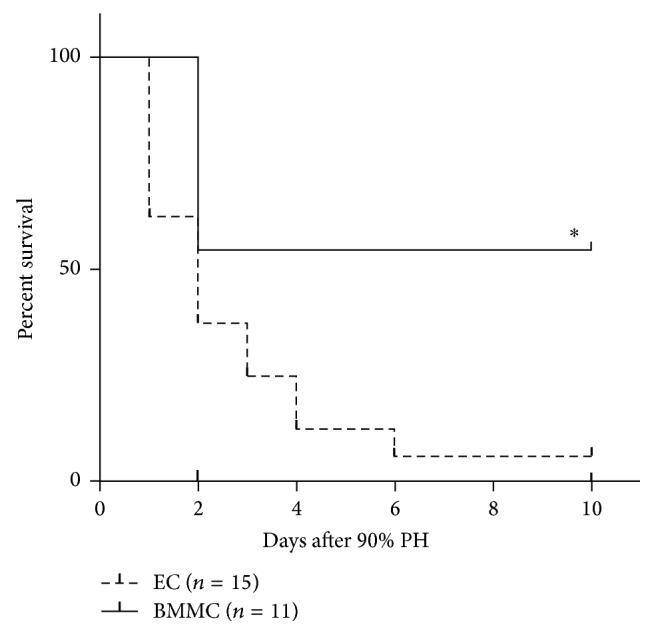
Survival rate for 10 days after 90% partial hepatectomy (PH). BMMC increase survival rate in rats submitted to PH (^*∗*^*P* = 0.003, log rank test). EC: empty capsules, BMMC: encapsulated bone marrow mononuclear cells.

Survival rate was accompanied during 10 days after 90% PH. The survival rate in BMMC group was higher (54.5%) than EC group (5%; *P* = 0.003; Figure 1). Interestingly, the peak of death in BMMC group occurred within the first three days after PH. In contrast, animals in EC group died over time. Thus, the next analyses were performed until 72 hours after 90% PH to address the beneficial effect of encapsulated BMMC.

### 3.2. BMMC Do Not Enhance Liver Regeneration

**Figure 2:**
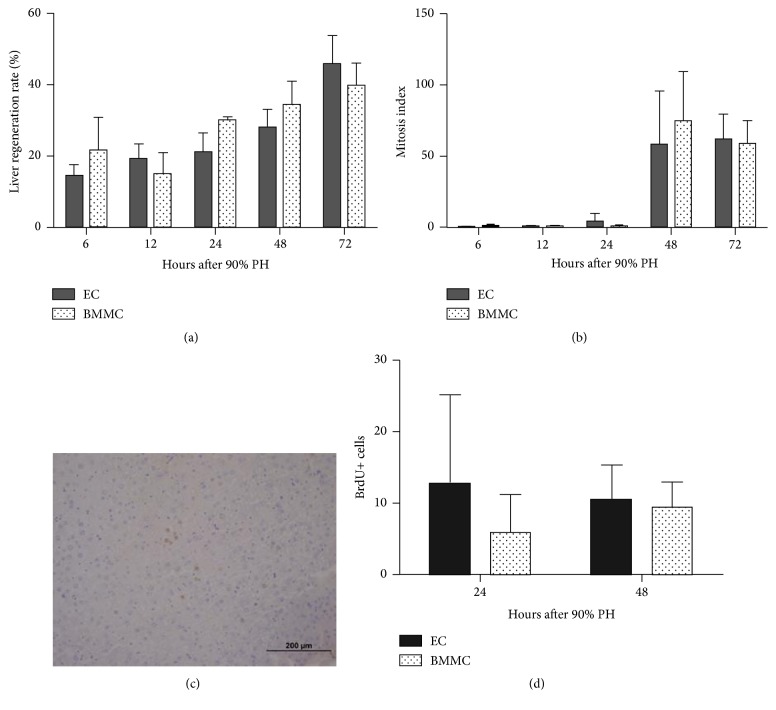
(a) Liver regeneration rate at 6, 12, 24, 48, and 72 hours after 90% partial hepatectomy (PH). (b) Mitotic index at 6, 12, 24, 48, and 72 hours after 90% PH. (c) BrdU immunohistochemistry, BMMC 48 hours after 90% partial hepatectomy (20x). (d) Positive hepatocytes for BrdU, EC, and BMMC 24: 12.72 ± 12.38 and 5.9 ± 5.3, respectively; EC and BMMC 48: 10.34 ± 5.03 and 9.46 ± 3.49, respectively. Values are expressed as means ± SD. Student's *t*-test. EC: empty capsules, BMMC: encapsulated bone marrow mononuclear cells.

To address if the increase of survival rate in BMMC was a consequence of a higher liver regeneration, we calculated liver regeneration rate until 72 hours after PH. Liver size increased over time in both groups and at 72 h the BMMC group reached 39% of the original liver weight, whereas EC group reached 46% (Figure 2(a)). In addition, the number of mitosis (Figure 2(b)) and positive hepatocytes for BrdU (Figures 2(c) and 2(d)) was also similar between groups. Therefore, BMMC do not seem to enhance liver regeneration after 90% PH.

### 3.3. BMMC Modulate Cytokines

**Figure 3:**
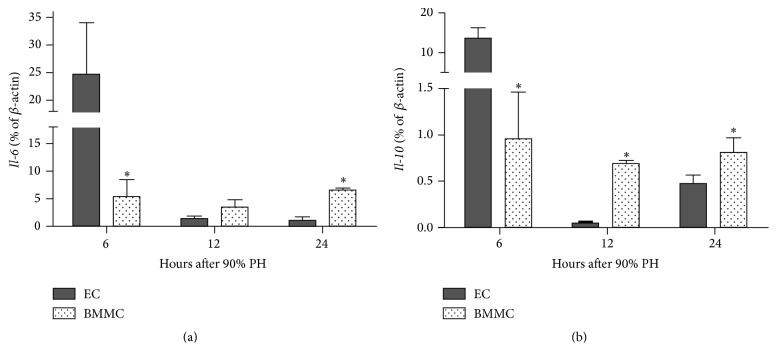
BMMC modulate liver cytokines. Liver gene expression of* Il-6* (a) and* Il-10* (b) at 6, 12, and 24 hours after 90% partial hepatectomy (PH). Values are expressed as means ± SD in log scale. Student's *t*-test, ^*∗*^*P* < 0.05. EC: empty capsules, BMMC: encapsulated bone marrow mononuclear cells.

It is well known that BMM have immunomodulatory properties [17]. Thus, in order to investigate the impact of immunomodulation on survival, we studied serum level of Interleukin-6 (IL-6) and its gene expression in the remnant livers. Serum IL-6 levels were increased in both groups at 6 h after 90% PH (268 pg/mL for EC group and 298 pg/mL for BMMC group, *P* = 0.9), but it decreased to normal range from 12 h in both groups equally (see Supplementary Figure  1 of the Supplementary Material available online at https://doi.org/10.1155/2017/5270527). However, in the remnant livers, we observed an increase in* Il-6 *expression in both groups until 24 hours after 90% PH compared to normal values. However, in BMMC group, this increase is lower (5-fold increase) at 6 hours when compared to EC group (25-fold increase, *P* = 0.03). From 12 h after PH, the expression in EC group decreased to about 2-fold normal values, whereas in BMMC it remained around 3–6 times higher than normal, albeit this difference is not statistically significant (Figure 3(a)).

On the other hand, for* IL-10* liver gene expression a diverse outcome was observed. At 6 h after 90% PH BMMC group showed near to normal expression values, whereas EC group showed a 12-fold increase compared to normal animals (Figure 3(b), *P* < 0.001 comparing BMMC versus EC groups). In the following hours (12 and 24 h after 90% PH) BMMC group remains close to normal values, whereas in EC group a sharp reduction in* IL-10* expression is observed (*P* < 0.001 and *P* = 0.02, resp.). At 48 and 72 h, though, BMMC show a reduction in expression.

### 3.4. Cytokine Modulation Is Not Related to the Number of Kupffer Cells

**Figure 4:**
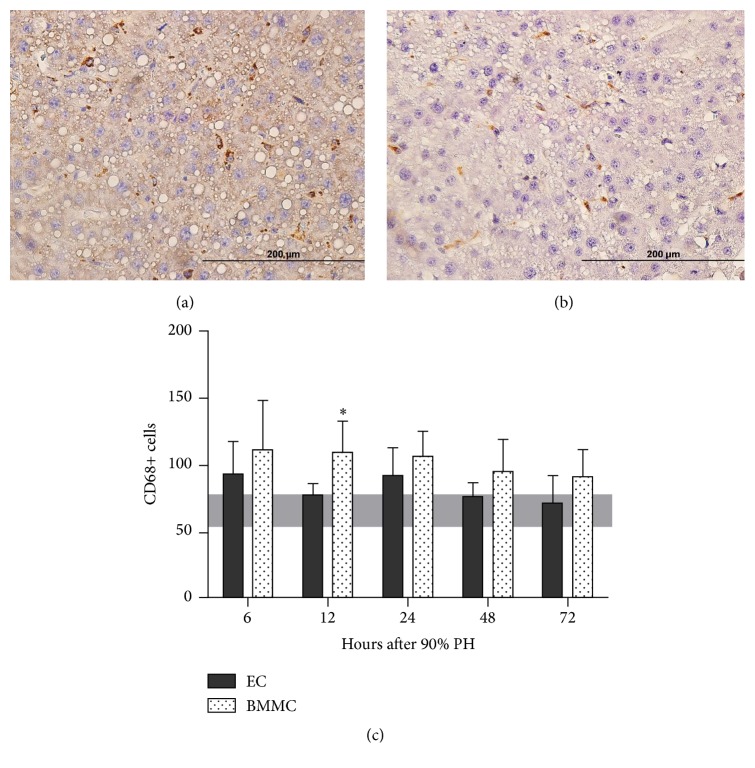
CD68+ cells in liver sections, EC, and BMMC 72 hours after 90% partial hepatectomy ((a) and (b), resp.). (c) CD68 + cell quantification at 6, 12, 24, 48, and 72 hours after 90% partial hepatectomy (PH). Values are expressed as means ± SD. Student's *t*-test, ^*∗*^*P* = 0.003. EC: empty capsules, BMMC: encapsulated bone marrow mononuclear cells. Horizontal bar indicates normal values.

Kupffer cells (KC) are the macrophages resident in the liver and have an essential role in liver injuries by secreting cytokines and priming hepatocytes for division. To address if the expression of cytokines in the liver were was related to the number of KC, we assessed the number of CD68+ cells in liver sections. We note that in general there is no difference in the amount of KC cells between the groups except at 12 hours, when BMMC group showed an increase when compared to EC group (*P* = 0.003; Figure 4).

### 3.5. BMMC Benefits Apoptosis

**Figure 5:**
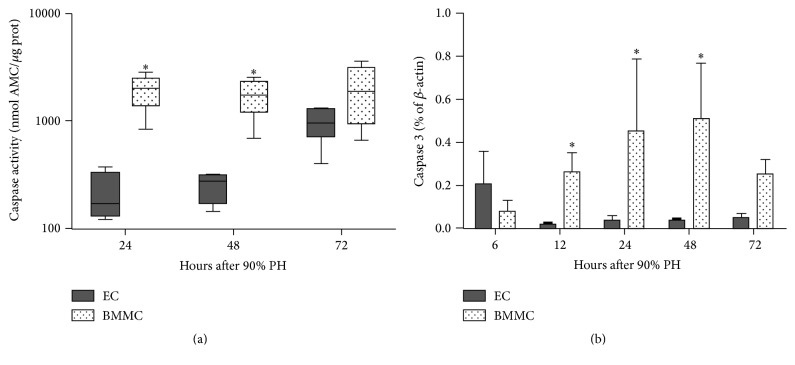
BMMC enhance apoptosis. (a) Caspase 3 activity and (b) liver gene expression of* Caspase 3* after 90% partial hepatectomy. Values are expressed as means ± SD. Student's *t*-test, ^*∗*^*P* < 0.05. EC: empty capsules, BMMC: encapsulated bone marrow mononuclear cells.

In a previous study [12], we showed that encapsulated whole bone marrow cells increase apoptosis after 90% PH. Therefore, we analyzed Caspase 3 activity and its expression in the remnant liver. It was observed that BMMC group showed more Caspase 3 activity mainly at 24, 48, and 72 h after 90% PH (*P* < 0.05; Figure 5(a)). This result is consistent with the gene expression data, which also was high in BMMC group compared to EC group (*P* < 0.05; Figure 5(b)).

## 4. Discussion

The use of BMMC in cell therapy is a suitable approach due to its easy standardized protocol for cell collection and promising benefits in the treatment of liver disease [5, 18]. However, before this therapy can be widely accepted in the clinic, its mechanisms of action must be better elucidated. Several studies have shown that BMMC have immunomodulatory properties [17, 19], focusing the attention of researchers on their paracrine effect [9]. In this sense, the use of microcapsules allows the communication between the cell entrapped within the capsule and the environment where they are implanted without cell-to-cell interaction [20]. In this study we evaluate the paracrine effect of BMMC using semipermeable microcapsules in rats with 90% PH, a suitable model for analyzing induced acute liver damage [21, 22].

The first result of our study showed a marked increase in survival rate in BMMC group which is consistent with other studies of cell therapy for acute liver disease using toxic models [6, 7, 23]. Other studies, using surgical models of 90% PH, such as Liu and Chang (2006) [24] and Uribe-Cruz et al. (2016) [12], also observed an increase in survival in animals treated with encapsulated cells, but both groups used encapsulated whole bone marrow cells, without separation of mononuclear cells.

In order to study the reasons for increased survival in BMMC group, we analyzed liver regeneration, which is a widely used method to assess recovery after partial hepatectomy [25, 26]. Liver regeneration rate, assessed either by liver weight, mitotic index, or BrdU, was similar in both groups suggesting that the increase in survival is not related to tissue regeneration. One must note that this is a 90% partial hepatectomy and not a classical liver regeneration model, such as 70% PH. So, although liver regeneration is essential for survival, different works point to other factors that influence recovery, including the pace of regeneration [27]. In addition, other studies from our group also point to a positive impact in survival that is not directly linked to hepatocyte regeneration [12, 28]. Thus, it is suggested that BMMC improve survival through mechanisms other than enhancing hepatocyte proliferation.

Acute liver failure promotes a shock septic response by the immune system [27, 28], leading to a disequilibrium in the levels of pro- and anti-inflammatory cytokines [29]. It is well recognized that IL-6 and IL-10 have an important role in improving recovery after acute liver failure [29, 30]. Thus, we analyzed the paracrine effect of BMMC on hepatic gene expression of* Il-6* and* Il-10* after liver injury. We observed that both experimental groups showed an increase in* Il-6* expression at 6 h after 90% PH; however in BMMC group this increase was much lower than in EC group. On the other hand, in BMMC-treated animals* Il-6* expression remains slightly elevated until 24 h after 90% PH. The expression of* Il-10* in BMMC group was also closer to normal levels, whereas in EC group it was increased at 6 h and greatly reduced at 12 hours after 90% PH. It is important to notice that* Il-6* is a major inducer of hepatic acute phase response and its expression plays a central role in restoring normal hepatic function following liver injury [31]. On the other hand,* Il-10* is a potent anti-inflammatory cytokine and selectively blocks proinflammatory genes in the liver after PH and reduces the number of macrophages and monocytes in the liver [32]. Moreover, the prevalence of an anti- or proinflammatory response results in the loss of immunohomeostasis and death [33]. So it can be suggested that the effect of BMMC is maintaining the balance between pro- and anti-inflammatory cytokines.

To address if the expression of cytokines in the liver was related to the number of KC, we assessed the number of CD68+ cells in liver sections. Normal Wistar rats have between 50 and 70 CD68+ cells, and this number does not increase significantly after 90% PH, except at 12 h, when BMMC show an average of 110 CD68+ cells. This lack of statistical difference may be due to the high variability observed among animals in both groups at any time point.

There are some works showing that these cytokines stimulate apoptosis in liver cells [34]. Our results show an increase in Caspase 3 activity and gene expression in BMMC group when compared to EC group at 24 and 48 hours. These results are in agreement with the suggestion that apoptosis eliminates unwanted or harmful cells to maintain homeostasis and normal tissue functioning [35]. It is a pathophysiological beneficial process that regulates growth and proliferation, thus ensuring proper organ size and function [36]. We are suggesting that the death of damaged hepatocytes by apoptosis may be a positive effect. There are studies showing that caspases can induce proliferation of neighboring surviving cells to replace dying cells in a process referred to as “apoptosis-induced proliferation” that may be critical for stem cell activity and tissue regeneration [35]. It is possible that such impact on proliferation occurred at later times, that is, after 72 hours, and therefore was not detected in our study. In addition, the death of damaged cells by apoptosis involves less cell leakage and recruitment of inflammatory cells [37] and the therapeutic modulation of apoptosis may represent a valid strategy for the treatment of human liver diseases [38]. Thus, the paracrine effect of BMMC induces Caspase 3, which may result in proper balance between cell death and division during liver regeneration.

## 5. Conclusions

In summary, BMMC regulate immune response and promote apoptosis in the liver after 90% PH by paracrine factors. These changes ultimately may be related to the higher survival observed in treated animals. Therefore BMMC are a promising alternative to treat acute liver failure.

## Supplementary Material

Serum level of Interleukin 6 (IL-6) was measured in blood collected at the time of death in rats receiving empty capsules (EC group) or capsules containing bone marrow mononuclear cells (BMMC group) after 90% partial hepatectomy (90% PH). Serum level of IL-6 was quantified by enzyme-linked immunosorbent assay (ELISA) using commercial kits (R&D Systems, Minneapolis, Minnesota, EUA) in accordance with the manufacturer's instructions. Both groups showed a significant increase of IL-6 at 6h post 90%PH, with progressive reduction to normal range after 12 h. No statistical difference was observed between groups.
