# Supplementary figures and images for: Paracrine Effects of Bone Marrow Mononuclear Cells in Survival and Cytokine Expression after 90% Partial Hepatectomy
Source: Stem Cells Int. 2017 Feb 23;2017:5270527. doi: 10.1155/2017/5270527 (PMC5343266; doi:10.1155/2017/5270527)

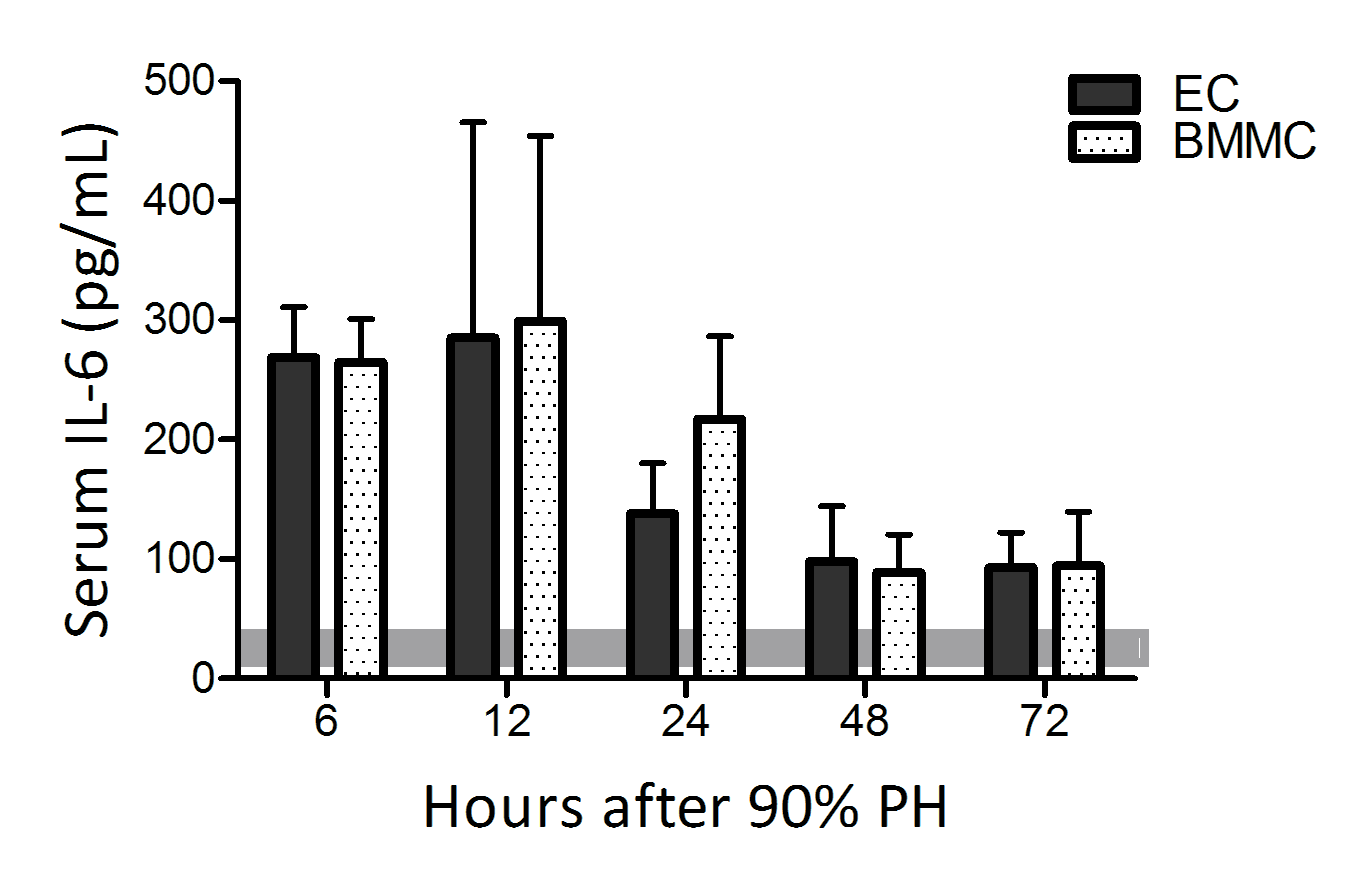

Supplement: Supplementary file 1 — Serum level of Interleukin 6 (IL-6) was measured in blood collected at the time of death in rats receiving empty capsules (EC group) or capsules containing bone marrow mononuclear cells (BMMC group) after 90% partial hepatectomy (90% PH). Serum level of IL-6 was quantified by enzyme-linked immunosorbent assay (ELISA) using commercial kits (R&D Systems, Minneapolis, Minnesota, EUA) in accordance with the manufacturer's instructions. Both groups showed a significant increase of IL-6 at 6h post 90%PH, with progressive reduction to normal range after 12 h. No statistical difference was observed between groups. [file 5270527.f1.tif]
